# Assigning Robust Default Values in Building Performance Simulation Software for Improved Decision-Making in the Initial Stages of Building Design

**DOI:** 10.1155/2015/246718

**Published:** 2015-05-18

**Authors:** Kyosuke Hiyama

**Affiliations:** ^1^Faculty of Engineering, Yamaguchi University, 2-16-1 Tokiwadai, Ube-shi, Yamaguchi 755-8611, Japan; ^2^Massachusetts Institute of Technology, Cambridge, MA 02139, USA

## Abstract

Applying data mining techniques on a database of BIM models could provide valuable insights in key design patterns implicitly present in these BIM models. The architectural designer would then be able to use previous data from existing building projects as default values in building performance simulation software for the early phases of building design. The author has proposed the method to minimize the magnitude of the variation in these default values in subsequent design stages. This approach maintains the accuracy of the simulation results in the initial stages of building design. In this study, a more convincing argument is presented to demonstrate the significance of the new method. The variation in the ideal default values for different building design conditions is assessed first. Next, the influence of each condition on these variations is investigated. The space depth is found to have a large impact on the ideal default value of the window to wall ratio. In addition, the presence or absence of lighting control and natural ventilation has a significant influence on the ideal default value. These effects can be used to identify the types of building conditions that should be considered to determine the ideal default values.

## 1. Introduction

Just as the implementation of high-efficiency HVAC and lighting systems and the use of natural energy are necessary in green building design, a well-designed architectural plan is important to minimize energy use in the building. Computer simulation tools, such as energy simulation tools, can be very useful in the development of such an optimal architectural plan [1]. For example, energy simulation tools can be useful in producing an enhanced sketch of the building shape that best minimizes energy consumption in early stages of the building process [2].

The implementation of energy simulation tools at the early stages of building design has high value in view of energy saving. However, energy simulation tools require several inputs. It makes energy simulation a time-consuming task [2]. In addition, the numerous uncertainties in these inputs make it difficult to perform an energy simulation efficiently in the real design process, especially when determining the building shape in the early stages of building design [3]. Information based on simulations with poor choices for the inputs may provide inaccurate information for decision making. It may also cause poor building performance.

How to handle the uncertainties is also an important topic in building information modeling (BIM [4]) technology development [5]. Based on this philosophy, the author has been developing an optimal building design aid system that integrates computer aided design (CAD), building environmental simulation tools and an optimization algorithm, based on the concept of BIM [6]. As an answer to these discussions, a new method assigning default values based on the past project records in building performance simulation software has been proposed [7]. The default values are defined as tentative values that are required in simulations in the initial stages of building design. The usage reduces the errors in building environment assessments by increasing the robustness of the building performance simulation results. In this paper, a more convincing argument is presented to utilize the new method to assign appropriate default values to a new building project.

## 2. Definition and Importance of Default Values at Early Stages of Building Design

The building shape has a large impact on the energy consumption of the HVAC and lighting systems. Thus the building shape is assigned first priority in the design [8–10]. In this context, the optimal building shape in view of energy saving should be developed as early as possible in the initial design stage [11, 12]. The façade, including the window proportions and the window areas, might be dictated by the architectural style and the sketch ideas could be guessed at the same time when the building shape is studied. However, the suitability for the building performance is not validated at this stage. Thus, these parameters should be optimized at the next design stage [13]. In addition, façade composition including glazing type and thickness for external walls might be dedicated based on green building guidelines. However, the optimal figures for these parameters vary according to building layout including the orientation [14]. Thus, these parameters might be changed due to detailed thermal energy and economic analysis at the later design stages. In these contexts, the sketch of the building shape should be studied in conjunction with the uncertainties in the façade features.

To carry out an energy simulation at this early design stage, tentative values must be carefully chosen for the numerous inputs that have not yet been determined. Some studies have identified the lack of quality in the model data related to the uncertainty as one of the main issues preventing the effective adoption of BPS in industry. Despite the development of BIM technology, which can aid in the input of building geometry, tentative values are still necessary for building properties and all nongeometric data inputs, such as the window properties [15]. In this study, these tentative values with uncertainty are called “default values” [7]. The use of “default values” is an advantage in many simulation tools; however, input quality control is one of the missing features regarding usability [16]. For example, the building proportion is a design variable when the building shape sketch is being developed. Meanwhile, the other values such as the window properties and the wall compositions that are studied at a later stage must be treated as “default values.” However, the design variables should include not only the building shape but also those default values that affect the optimization outputs. That is, the optimal design value is highly affected by the default values. A poor choice of default values, therefore, can direct an architect toward ineffective building shapes, which can result in buildings with high energy consumption. In other words, a realistic design approach is to introduce ranges of uncertainty in the simulation parameters including the default values [17]. In particular, the window properties, such as the glazing percentage and the orientation, strongly influence the output [18]. Moreover, the building operation, for example, the implementation of natural ventilation, affects the optimal window properties [19]. Thus, thoughtful consideration is required in assigning default values to the window properties. The optimized design solutions must be robust enough to changes in the design conditions and to identify solutions that are less susceptible to uncertainty [20]. Thus, the demand of research for investigating the sensitivity of design condition is increasing [21].

To ensure the robustness of the optimized design solution, background data, such as the energy simulation results, should also be sufficiently robust to the uncertainties in the inputs, which may vary in subsequent design stages. Thus, the ideal default value should be defined to ensure robustness. The default values should comprise the values that maximize the robustness instead of the optimal values that minimize the object functions, such as energy consumption. Note that the default values are expected to be treated as design variables and will be optimized in a subsequent design stage.

It is difficult to find a global default value that can be applied to any type of building design. The optimal building design is strongly dependent on the climate [21]. Thus, building components that can be adapted to different climates should be employed.

The question then arises as to whether default values based on different climates can be developed, just as each country has its own green building guidelines. It may be possible to define appropriate default values for simple properties such as the glazing type. However, window properties such as the window geometry are strongly dependent on the building shape and the building operation [18]. That is, the original window properties of each building should maximize the energy efficiency based on the building shape and operation. Therefore, there are as many appropriate default values as there are varieties of buildings [7].

To assign appropriate default values to a new building project, an experienced architect searches for the best practice that shows similarities to the new project to minimize ranges of uncertainty. In a survey of current building simulation workflows in professional practice, 31% of the participants indicated that they reused a previous model for building information inputs [15]. An architect usually aims to determine the best practice that is suitable for a new project. BIM can help to reduce the effort expended by architects in searching for best practices.

**Figure 1:**
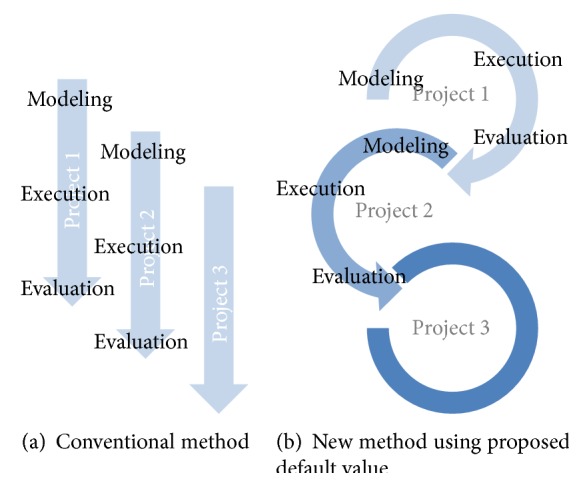
Image of simulation flow.

In BIM technology development, data-mining techniques are controversial [22]. However, there appears to be less debate on how to search for data from existing building data sets. The building information is represented as the assembly of objects in BIM that employs the object-oriented databases. Existing building objects are highly developed adaptations to previous building projects that account for the unique features of the building. These concepts can be used in knowledge transfer methods by employing the features of existing building parts to benefit a new building design that has similarities to the previous project [23]. In a previous paper, a method was presented for using building attributes for a new project that had been optimized using previous building designs [7, 24].  Figure 1 shows the image of the simulation process using a conventional method (Figure 1(a)), in contrast to the simulation process displayed in Figure 1(b), in which the default values are used that were proposed from previous studies.

Although all building properties are individually inputted using a conventional method (Figure 1(a)), the simulation inputs are transferred from the previous studies using proposed default values in the proposed simulation flow (Figure 1(b)).

In this paper, a more convincing argument is presented to show the significance of the new method. Case studies are performed to optimize window properties under various conditions for the building shape, climate, and building operation modes, such as lighting control and natural ventilation. First, the variation in the optimal window properties under different conditions is investigated. Then, the relationship between the optimal properties and each of the conditions is determined. These relationships are used to develop a strategy for searching for similarities between a new project and previous projects to determine appropriate “default values” for the new project.

This study focuses on window-to-wall ratio (WWR) for two reasons. The first reason is the significance of the impact on the simulation results. The sketches of the building shape and location are discussed at the beginning of building design. Building performance simulations are extremely important because the two factors are critical to the building performance. The default value of WWR is required. Another reason is the feasibility of the proposed method. The window data are easily output from the BIM data, such as the industry foundation classes (IFC) data model [25].

## 3. Case Study

### 3.1. Calculation Scheme

First, various optimal solutions under different conditions are determined. We consider a window design problem that is often used in case studies for optimization research [26]. A three-story office building model is used in this case study. Several properties of this building model can be set parametrically, so that the influence of default values can be properly investigated. One of these properties is the floor area of one floor, which can be set to 400 or 1600 m^2^. In this study, only the second floor is considered to determine the building output. The floor height is 3.5 m, including the plenum space.

**Figure 2:**
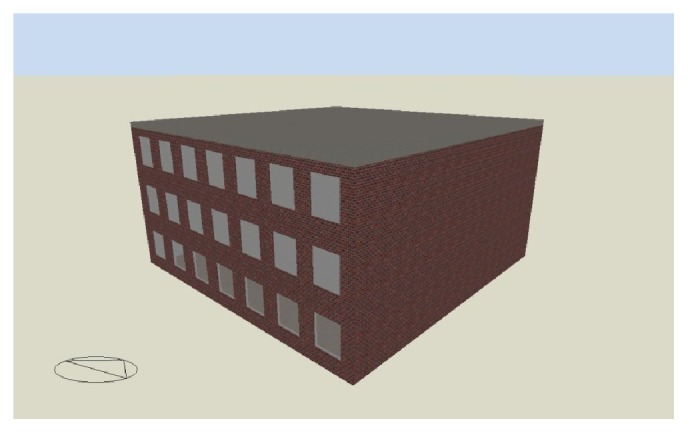
Building object.

Figure 2 shows the building object with a floor plan of 400 m^2^. The building object with an area of 400 m^2^ is regarded as a small-scale building with a small space depth from the window, whereas the building object with an area of 1600 m^2^ is regarded as a large-scale building with a large space depth. The design variable for the optimization is the window-to-wall ratio (WWR), which strongly affects the energy use of a building. The glazing is generated for a window height of 2.0 m, but the window height can be adjusted to achieve the required WWR. The objective function is the annual CO_2_ emission.

Case studies are conducted based on energy simulations for various conditions, building sizes, orientations, building operation modes, such as lighting control and natural ventilation, and weather data. The weather data for ASHRAE/TMY3 in Boston, MA, USA, are chosen to represent a subarctic climate, and the weather data for ASHRAE/TMY3 in Miami, USA, are chosen to represent a subtropical climate.

**Figure 3:**
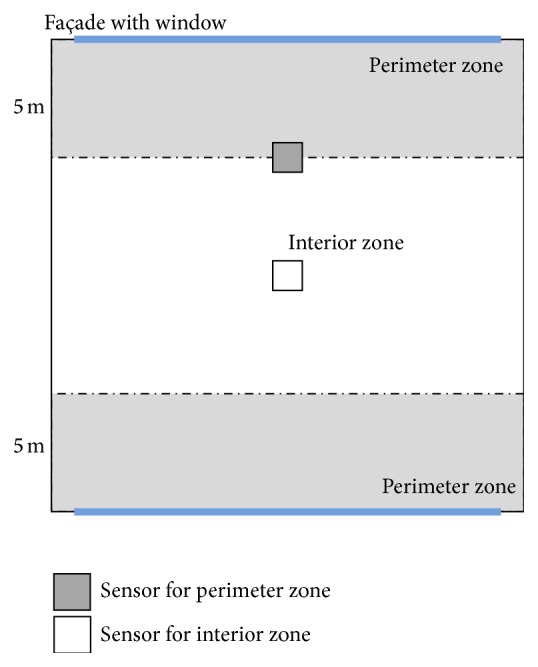
Sensor locations.

Lighting control depending on daylight use is simulated for the building operation. The office area is separated into two zones: a perimeter zone and an interior zone. The perimeter zone is the area that falls within a 5.0 m distance from a wall with windows. Each zone contains an illuminance sensor. This sensor is located at the center of the office in the interior zone. In the perimeter zone, the sensor is located at the boundary between the two zones. The sensor locations are shown in Figure 3.

Natural ventilation that depends on the external air temperature is simulated. The windows open when the interior air temperature is higher than both the external air temperature and the set point temperature. The set point temperature is 24°C. The window openings are modulated by the temperature difference between the interior and the exterior. The maximum opening area is 20% of the window area. This opening area is multiplied by a factor from 0 to 1. When the temperature difference between the interior and the exterior is less than 4°C, this factor is 1. The factor decreases linearly as the temperature difference increases and becomes 0 for a temperature difference of 8°C. The ventilation is cross ventilation. The ventilation rate through each opening is calculated based on the pressure difference using wind pressure effect.

**Figure 4:**
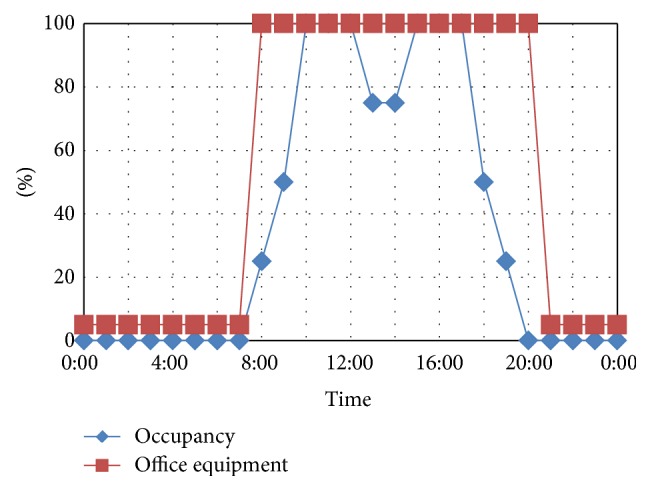
Schedule for internal heat load.

The variable conditions for the case studies are shown in Table 1. The remaining conditions are the same for each case, as shown in Table 2. The activity schedules are shown in Figure 4. Design Builder [27] integrating Energy Plus [28] simulation engine were used for the simulations. Only the energy consumption of the second floor is considered in these case studies.

### 3.2. Calculation Results (Boston)

**Figure 5:**
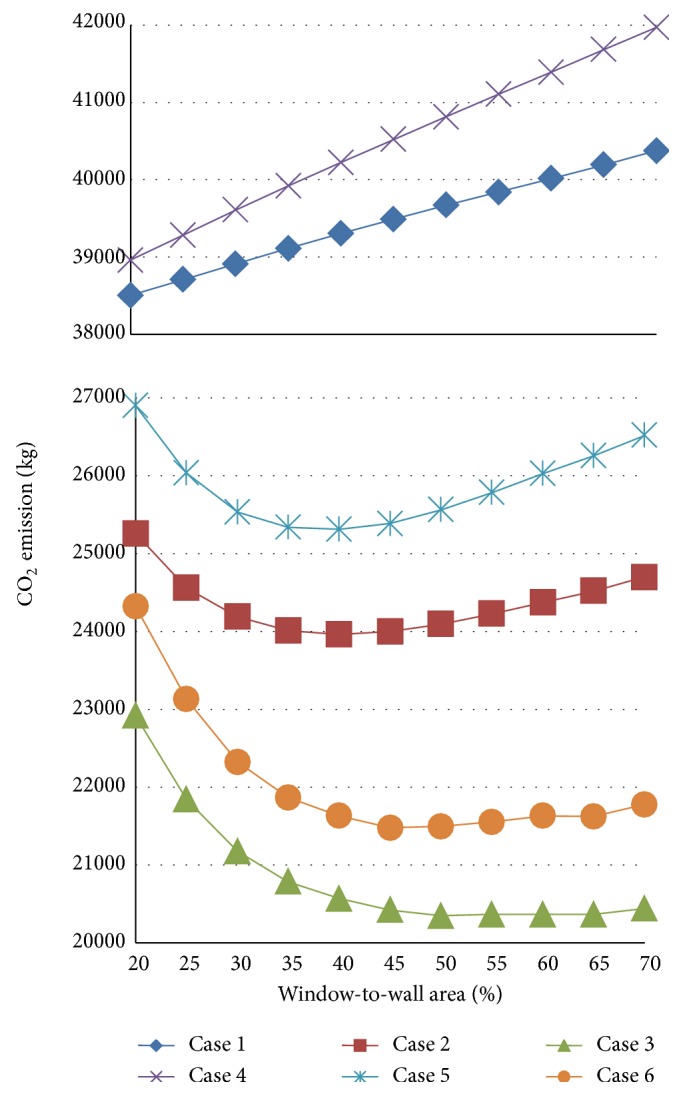
Results for cases 1–6.

Figure 5 shows the calculation results for cases 1–6, using the weather data from Boston for a floor area of 400 m^2^. In cases 1 and 4, neither the lighting control nor the natural ventilation control are simulated. In these two cases, the larger the WWR, the higher the CO_2_ emissions. The cooling load increases with the window area due to the increase in solar gain and heat transmittance through the window.

In cases 2 and 5, daylight-dependent lighting controls are simulated. In these two cases, the CO_2_ emissions are clearly reduced by using lighting control in comparison to cases 1 and 4, in which lighting control is not used. Increasing the window area increases the potential for daylight use. In both cases, this effect is clearly shown for a WWR of approximately 40%. However, this daylight effect saturates when the WWR exceeds 50%. Moreover, the CO_2_ emissions increase with the window area. The results seem to be comparable to other evaluation results in references [29] and the ANSI/ASHRAE/IESNA1 Standard 90.1-2010, which attained a maximum WWR of 40%. Thus, increasing the window area beyond 50% of the wall area is not effective for reducing CO_2_ emissions.

In cases 3 and 6, natural ventilation and lighting control are simulated. In these two cases, the effect of cooling by natural ventilation and lighting control can be clearly observed when the WWR reaches 50%. Beyond this value, the window areas have a small influence on CO_2_ emissions. Although the CO_2_ emission rates for cases 4–6, in which the windows face east and west, tend to be larger than those for cases 1–3, in which the window orientations are north and south, similar trends of increasing CO_2_ emissions are obtained for larger window areas.

**Figure 6:**
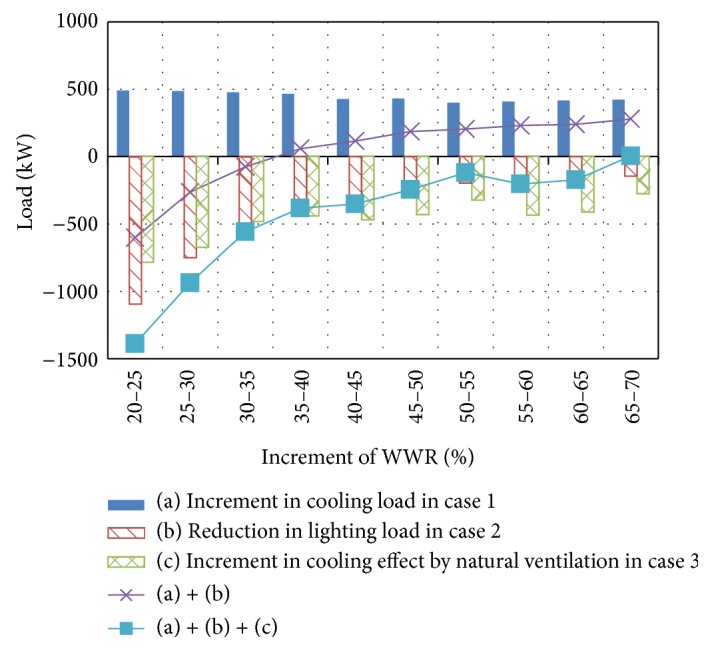
Influence of window area.

Figure 6 shows the influence of the window area on the cooling load for case 1, the lighting load reduction achieved with lighting control for case 2, and the cooling effect of natural ventilation in case 3 (see (a), (b), and (c) in Figure 6, resp.). The cooling load in case 1 increases monotonically with the window area. However, the lighting load reduction in case 2 decreases as the window area increases. For the cases in which only lighting control is activated, cases 2 and 5, the lighting load reduction achieved by lighting control is larger than the increase in the cooling load obtained by increasing the window area up to a certain WWR. An inflection point is then reached at which the lighting load reduction becomes equal to the HVAC load increment (see (a) + (b) in Figure 6). The inflection point occurs at a WWR of 40% in this case study. Thus, a single optimal WWR can be determined. The optimal WWR is approximately 40% in cases 2 and 5. As shown in Figure 6, the cooling effect of natural ventilation increases almost monotonically with the window area. For the cases in which both lighting control and natural ventilation are simulated, cases 3 and 6, the CO_2_ emission decreases until the glazing percentage reaches approximately 50%, as found for cases 2 and 5. The influence of the glazing percentage on the CO_2_ emissions becomes very low because lighting control has a very small effect, whereas the constantly increasing cooling effect of natural ventilation is nearly equal to the increase in the cooling load obtained by increasing the window area (see (a) + (b) + (c) in Figure 6).

**Figure 7:**
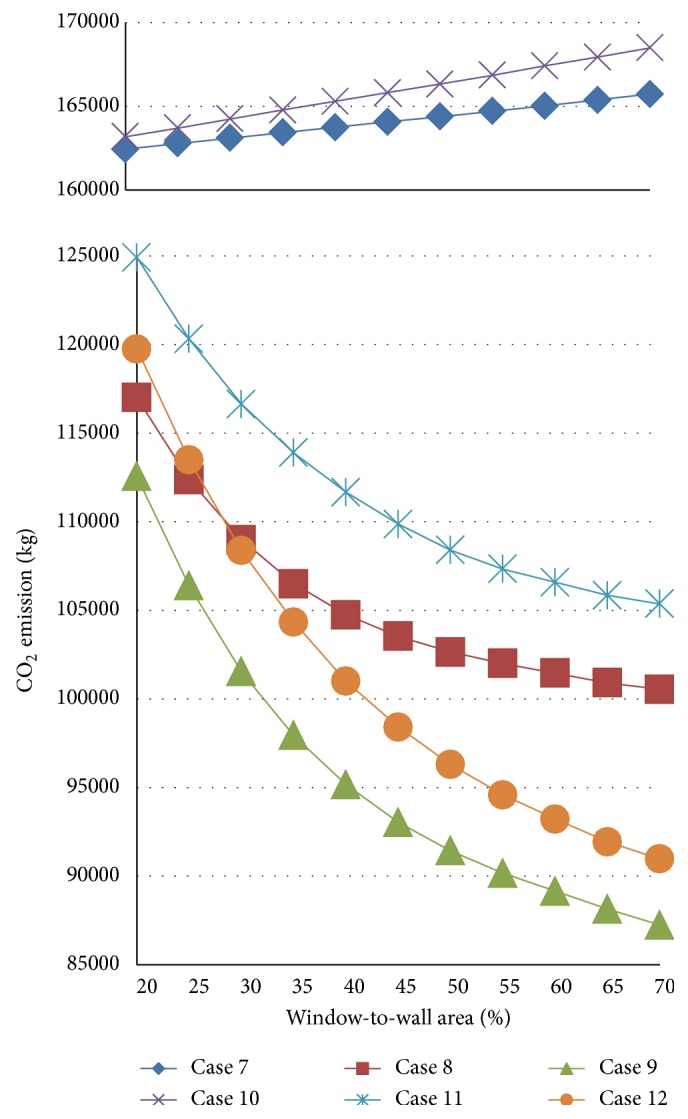
Results for cases 7–12.

Figure 7 shows the results for cases 7–12, which have floor areas of 1200 m^2^, and the space depth is large. In cases 7 and 10, neither lighting control nor natural ventilation control are simulated. In these two cases, the larger the WWR, the higher the CO_2_ emission, as was found for cases 1 and 4. However, as the window area increases, the CO_2_ emission monotonically decreases for all of the other cases. Thus, daylight use achieved by increasing the window area is always expected to reduce CO_2_ emissions, even though the influence gradually decreases. Thus, the design with the highest WWR is the best solution for cases with a relatively large floor area and large space depth.

### 3.3. Calculation Results (Miami)

**Figure 8:**
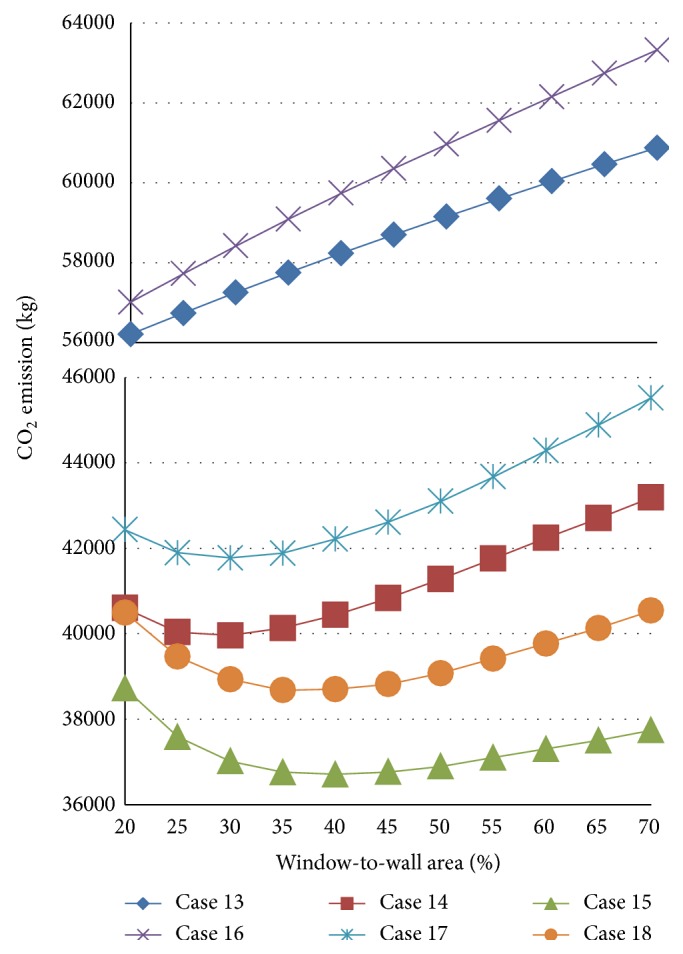
Results for cases 13–18.

Figure 8 shows the results for cases 13–18, based on the weather data from Miami for a floor area of 400 m^2^. Compared to Figure 5, the same trends are observed for the influence of the window area on the CO_2_ emissions. However, the inflection points of the CO_2_ emission curve are more obvious than those observed in Figure 5. Although it is difficult to find an optimal WWR in cases 3 and 6, a window area of 40% can be used as the optimal percentage in cases 15 and 18.

**Figure 9:**
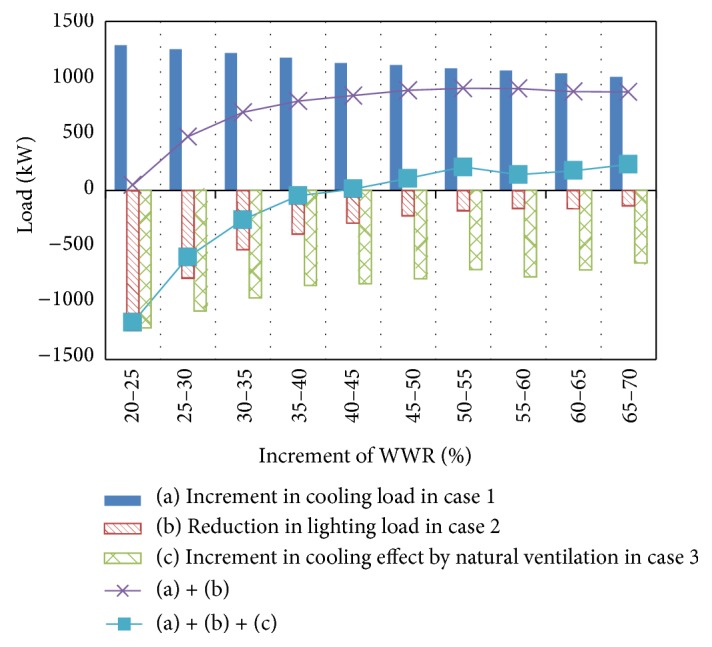
Influence of window area.

Figure 9 shows the influence of the window area on the cooling load for case 13, the lighting load reduction for case 14, and the cooling effect due to natural ventilation in case 15 (see (a), (b), and (c) in Figure 9, resp.). The trends shown in Figures 9 and 6 differ in that the increase in the cooling load is relatively larger than those for the lighting load reduction and the cooling effect by natural ventilation. Thus, when the WWR reaches an inflection point, the lighting load reduction becomes equal to the HVAC load increment (see (a) + (b) in Figure 9). Even for natural ventilation operation, the increment in the cooling load becomes larger than the sum of the lighting load reduction and the cooling effect as the window area increases. Thus, an inflection point can be observed even for the case with natural ventilation (see case 15 in Figure 8 and (a) + (b) + (c) in Figure 9).

**Figure 10:**
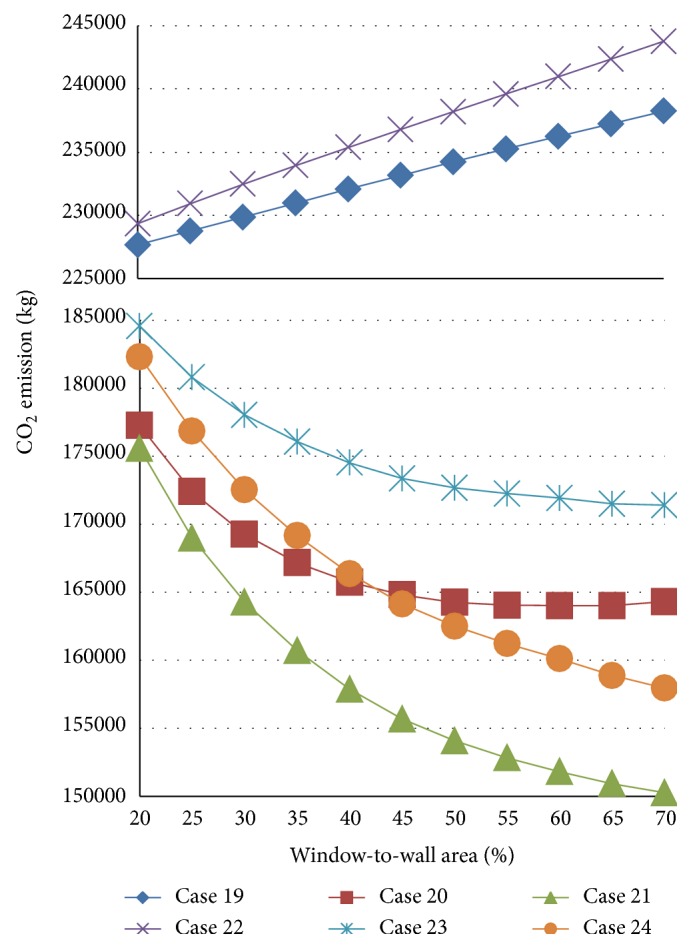
Results for cases 19–24.

Figure 10 shows the results for cases 19–24, which have floor areas of 1200 m^2^. In comparison to Figure 5, the same trends are observed for the influence of the window area on the CO_2_ emissions. Thus, the weather data do not exert a significant effect on the optimal window area for cases with a relatively large floor area and a large space depth from window. A reduction in CO_2_ emissions is expected because the increased window area contributes to a lighting load reduction and the possibility of using natural ventilation in all of the cases considered.

## 4. Discussion

The ideal “default values” referenced in the “Introduction” are discussed in this section. When a building shape sketch is discussed at the early stage of building design, an energy simulation must be implemented to estimate the energy performance of the building. In energy simulations, the window properties, especially the WWR, exert a strong effect on the output. Thus, accurate inputs are needed to obtain an optimal building shape that maximizes the energy performance of the building. However, the window properties are generally not considered at this stage because the building shape sketch is usually considered at the first stage of the building design. Thus, tentative values should be assigned to the window properties to implement the energy simulation. However, these tentative values will be changed at a later stage of building design, when the window properties are treated as design variables. Thus, one must choose a tentative value that minimizes the variation in the calculation results when the value is changed at a later stage in the building design.

### 4.1. Evaluation of Default Value Using Average Value of Error

**Figure 11:**
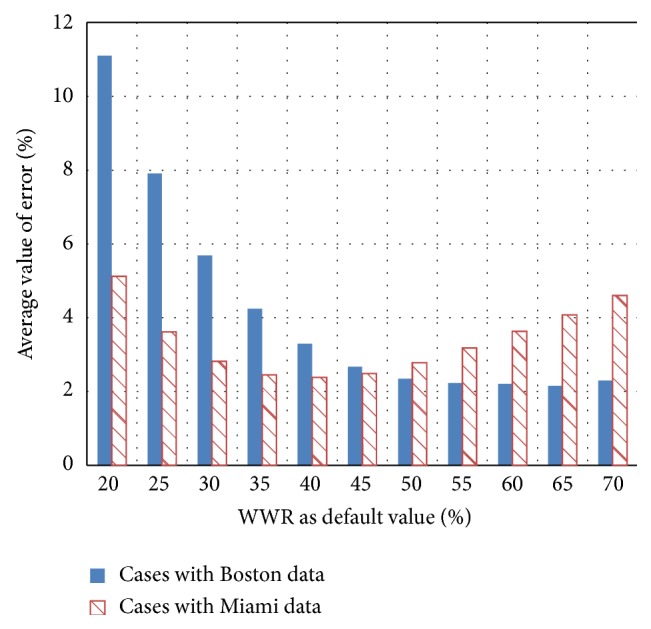
Average value of the error.

We illustrate this concept using the case study presented in Section 3.  Figure 11 shows the average value of the error when different WWRs are used as tentative values. The average values of the error are calculated using the following equation:

(1) $$\begin{array}{c} E\left(i\right)=\overset{}{\underset{j}{\sum }}\frac{Q_{i,j}-Q_{\min,j}}{Q_{\min,j}}\times \frac{100}{n}, \end{array}$$

*E*(*i*) is average value of the error [%],  *Q*_*i*,*j*_ is CO_2_ emission for case *j* when the tentative WWR is *i*% [kg], *Q*_min⁡,*j*_ is minimum CO_2_ emission for case *j* [kg], and *n* is total number of cases used to calculate the average value.

The error *Q*_*i*,*j*_ − *Q*_min⁡,*j*_ is the amount of change that results from the change in the default value at a subsequent stage in the building design. The amount of change is divided by the minimum CO_2_ emission *Q*_min⁡,*j*_ as the calculation results using the optimal WWR to express the value as a percentage. The percentage is assumed to be the error range in the simulation results in the initial stages of building design. Minimizing the value of this change maintains the accuracy of the simulation results in the initial stages of building design. In this equation, each value is assumed to have an equal probability of being selected as the default value and the default value is assumed to be optimized to reduce the CO_2_ emission at a subsequent stage in the building design.

The Boston data are used in the calculations for cases 1–12, and the Miami data are used in the calculations for cases 13–24. For the cases in which the Boston data are used, the average value of the error is minimized for a WWR of approximately 65%. Thus, a relatively large window area can be used as the ideal default value in a subarctic climate area such as Boston. However, for the Miami cases, the average value of the error is minimized for a certain WWR, such as 40%. Thus, expanding the window area is not necessarily a good strategy for reducing CO_2_ emissions by daylight use and natural ventilation. A modest WWR should be used as the ideal default value for a subtropical climate area such as Miami.

### 4.2. Grouping of Cases to Increase Adequacy in Default Value

**Figure 12:**
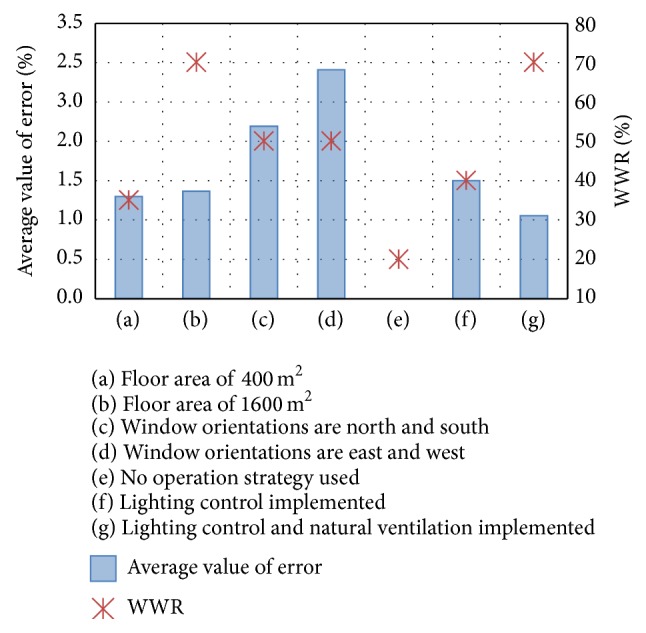
WWRs that produce a minimum average value of the error.

Figure 12 shows the WWRs that produce the minimum average value of the error, grouped by cases with a common building feature. Figure 12(a) shows the results for the cases with a floor area of 400 m^2^, cases 1–6 and cases 13–18. An optimum WWR of 30% should be used as the default value for this condition. Figure 12(b) shows the results for cases with a floor area of 1600 m^2^, cases 7–14 and cases 19–24. In this scenario, an optimum WWR of 70% should be used as the default value, corresponding to twice the value found for a floor area of 400 m^2^. This result implies that the default values should be selected based on the space depth related to the floor area. If the building scale is large and the space depth is large, as in case (b), a relatively large ideal default value should be used. The effects of daylight use and natural ventilation can be expected for larger window areas. However, if the building scale is small and the space depth is small, as in case (a), a modest WWR should be used as the ideal default value.

Figures 12(c) and 12(d) show the results for cases in which the window orientations are north/south and east/west, respectively. In both cases, modest WWRs, near 50%, result in the minimum average value of error. Thus, the window orientation has little effect on the optimal default value.

Figures 12(e), 12(f), and 12(g) show the results for cases with different operation modes of lighting control and natural ventilation. If neither operation is employed, as in case (e), the window area should be minimized because increasing the window area directly increases the HVAC load. However, when lighting control is used, increasing the window area is an appropriate strategy for reducing CO_2_ emissions. Then, a modest WWR can be used as the default value. When both lighting control and natural ventilation are utilized, an increased window area tends to reduce CO_2_ emissions. In this case, a large window area should be used as the default value. The installation of a ventilation tower could also be considered as a default input for an energy simulation because the natural ventilation effect does not saturate, even at the highest WWR. These results imply that the presence or absence of these operation modes must be included at the early stage of building design to increase the precision of the simulation at this stage.

## 5. Conclusions

To minimize the magnitude of the change in the default values, the default values should be chosen in accordance with the building design conditions, such as the building scale, location, and operation mode, including lighting control and natural ventilation. In this study, we first confirm that the ideal default values depend on the building design conditions. When a building scale and space depth are relatively small, each case has a different optimal WWR. The optimal window sizes vary depending on the climate condition and the building operation mode. However, for buildings with relatively large scales with large space depths, an increase in the window area is an appropriate strategy for any climate condition.

Then, we investigate the influence of each condition on the variation in the ideal default values for the WWR. These effects can be used to determine the types of building conditions that should be considered when selecting the ideal default values for a new building design project. In the case studies considered, the building scale and the space depth are found to have a large impact on the ideal default value of the WWR, whereas the window orientation has little impact. In addition, the presence or absence of lighting control and natural ventilation has a significant influence on the ideal default value. Thus, the operation mode must be chosen at the early stage of the building design because these elements are critical in determining the optimal window area.

This study focuses on the WWR because the value has a significant impact on the energy simulation results. The concept of an ideal default value can presumably be adapted to any input required for building performance simulations. In future studies, the author plans to apply the idea to other inputs, such as the inputs required for template files of standardized building properties [15].

## Figures and Tables

**Table 1:** Variable conditions for the case studies.

Case	Operation	Window orientation	Area of each floor [m^2^]	Weather data
1	None	North and South	400	Boston
2	Lighting control			
3	Lighting control, natural ventilation			
4	None	East and West		
5	Lighting control			
6	Lighting control, natural ventilation			
7	None	North and South	1600	
8	Lighting control			
9	Lighting control, natural ventilation			
10	None	East and West		
11	Lighting control			
12	Lighting control, natural ventilation			

13	None	North and South	400	Miami
14	Lighting control			
15	Lighting control, natural ventilation			
16	None	East and West		
17	Lighting control			
18	Lighting control, natural ventilation			
19	None	North and South	1600	
20	Lighting control			
21	Lighting control, natural ventilation			
22	None	East and West		
23	Lighting control			
24	Lighting control, natural ventilation			

**Table 2:** Simulation conditions.

*U* value	Outer wall: 0.25 W/m^2^K (concrete block and brickwork) Ground floor: 0.15 W/m^2^K, roof: 0.15 W/m^2^K Window: 1.96 W/m^2^K (double glazing)

Window	Double glazing, *U* value: 1.96 W/m^2^K, total solar transmission (SHGC): 0.70, direct solar transmission: 0.62, and light transmission: 0.74

Window shading	Blind with high reflectivity slats Solar setpoint for cases 1, 5, 9, 13: 120 W/m^2^ Maximum allowable glare index for the other cases: 22.0

Internal heat	Human: 0.1 person/m^2^, 123 W/person, office equipment: 11.8 W/m^2^

Lighting	3.3 W/m^2^—100 lux Target illuminance: 400 lx

Mechanical ventilation	10 L/s-person

Heating	Natural gas (carbon emission factor: 0.195 kg CO_2_/kWh) Heating system CoP: 0.830 Schedule: 5:00–19:00 in weekday: on, all other periods: on by set-back temp. Setpoint temperature: 22°C, set-back temperature: 12°C

Cooling	Electricity from grid (carbon emission factor: 0.685 kg CO_2_/kWh) Cooling system CoP: 1.670 Schedule: 5:00–19:00 in weekday: on, all other periods: off Setpoint temperature: 26°C
